# A case of hepatic intraductal papillary neoplasm of the bile duct

**DOI:** 10.1259/bjrcr.20210172

**Published:** 2021-11-16

**Authors:** Eisuke Mukaida, Akio Tamura, Masao Nishiya, Kenichi Katoh, Tamotsu Sugai, Kunihiro Yoshioka

**Affiliations:** 1 Department of Radiology, Iwate Medical University School of Medicine, Morioka, Japan; 2 Department of Molecular Diagnostic Pathology, Iwate Medical University School of Medicine, Morioka, Japan

## Abstract

In this report, we present a 57-year-old female with a history of mild alcoholic liver disease during a medical check-up. Abdominal computed tomography and magnetic resonance imaging showed a multicystic mass with a solid enhancing mural nodule in the right lobe of the liver. Subsequently, laparoscopic right liver lobectomy was performed and pathological findings revealed intraductal papillary neoplasm of the bile duct (IPNB) with an associated invasive carcinoma. IPNB is a relatively rare disease that should be considered in the differential diagnosis of hepatic cystic tumours. Our case report highlights the importance of capturing image findings of the IPNB as this disease has a high potential for malignancy.

## Clinical presentation

A 57-year-old female with a history of mild alcoholic liver disease underwent a medical check-up at her company. Initial non-enhanced abdominal computed tomography (CT) revealed a large cystic mass in the right lobe of the liver. Laboratory findings revealed elevated serum levels of tumour markers CA19-9 (373 U ml^−1^) and CEA (23.6 ng ml^−1^). She was referred to our hospital for further investigation, and contrast-enhanced CT and Gadolinium (Gd)-enhanced magnetic resonance imaging (MRI) were performed.

## Differential diagnosis

Intraductal papillary neoplasm of the bile duct (IPNB), mucinous cystic neoplasm (MCN) and other hepatic cystic diseases (*e.g.,* peribiliary cyst, simple biliary cyst and complicated cyst) were suspected.

## Imaging findings

**Figure 1.**
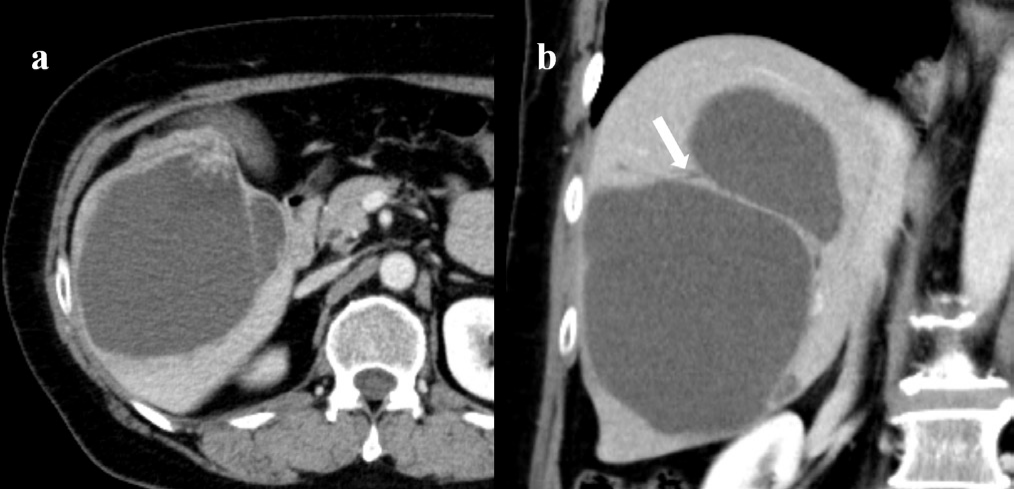
Abdominal contrast-enhanced computed tomography (venous phase). (**a**) Axial image shows multicystic mass with a solid enhancing mural nodule in the right lobe of the liver. (**b**) Coronal image shows the mass connecting with B7 (arrow) with slightly upstream dilatation

Contrast-enhanced abdominal CT showed a multicystic mass 115 × 81 × 130 mm in maximum TR × AP × CC (transverse × anteroposterior × craniocaudal) with a solid enhancing mural nodule in the right lobe includes segments 5, 6 and 7 of the liver (Figure 1a). There was thick septation and no peripheral calcification in the mass. This study also revealed the mass connecting with intrahepatic bile duct (B7) with a slightly upstream dilatation (Figure 1b). Contrast-enhanced CT showed no evidence of distant metastasis.

**Figure 2.**
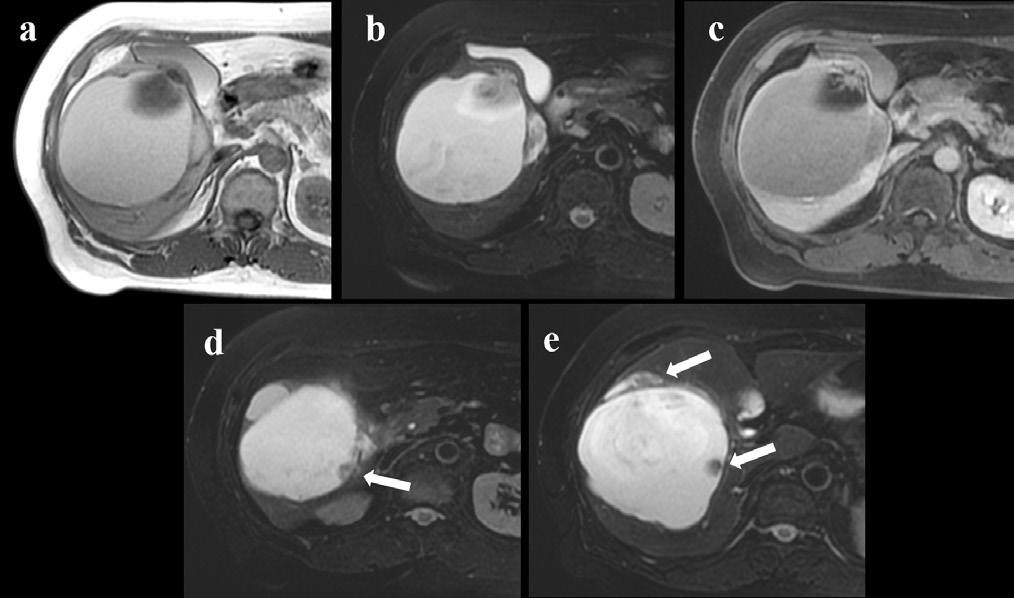
Abdominal magnetic resonance (MR) imaging. (**a**) Axial T1-weighted image shows the cysts of high intensities, suggestive of the presence of a mucin-containing tumour. (**b**) Axial T2-weighted fat suppressed image shows cysts with septation and the mural nodule. (**c**) Axial Gd-enhanced T1-weighted image shows an enhanced mural nodule. (**d–e**) Axial T2-weighted fat suppressed image shows several mural nodules in the cystic mass (arrows)

On T2-weighted and T1-weighted MRI, we observed the mass with a slightly high signal intensity (Figure 2a,b). These imaging findings suggest that the mass contained mucin. Gd-enhanced MRI was performed to confirm an enhanced mural nodule, and T2W fat suppressed imaging also showed mural nodules in the mass (Figure 2c–e). MRI did not clearly show communication between the mass and the intrahepatic bile ducts. Based on the laboratory test and radiological findings, a diagnosis of malignant neoplasm of the liver was established. Surgery was suggested, but she initially disagreed. Five months later, a repeated MRI revealed a slightly enlarged mural nodule. There were no findings indicating metastasis on the CT image, except that finding of the right lung.

## Treatment/Outcome/Follow-up

**Figure 3.**
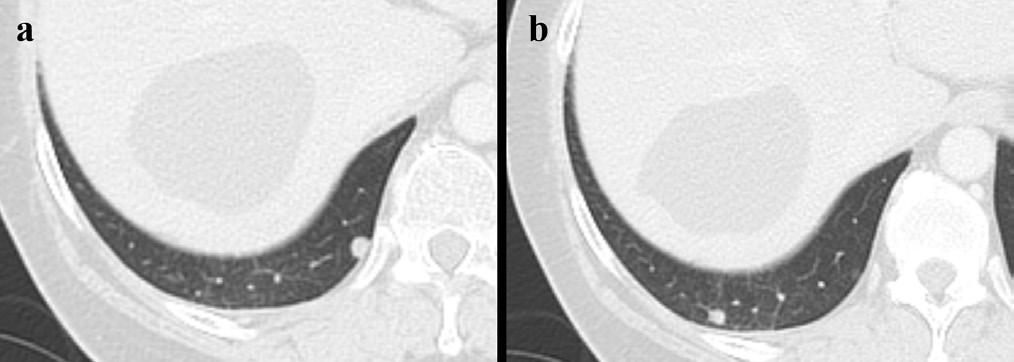
Chest computed tomography performed 5 weeks postoperatively. (a, **b**) Axial image shows two nodules in the lower lobe of the right lung (each nodule size is 5 mm)

A discussion with the patient regarding further management was undertaken, and finally, she agreed to the surgery. She had a history of mild alcoholic liver disease, but her Child-pugh score was 5 and her preoperative indocyanine green retention rate at 15 min (ICG-R15) was 8.7%. Therefore, she was able to undergo the right hepatic lobectomy without lymphadenectomy. No major postoperative complications were observed. The patient was discharged 13 days after the surgery. A CT scan performed 5 weeks after the surgery revealed two nodules in the lower lobe of the right lung, and the patient was diagnosed with lung metastases (Figure 3). For treatment, the patient was prescribed gemcitabine and carboplatin therapy. CT performed 4 months postoperatively showed increased lung metastasis and paraaortic lymph nodes metastasis. Chemotherapy was changed to gemcitabine and TS-1 therapy.

## Pathological findings

**Figure 4.**
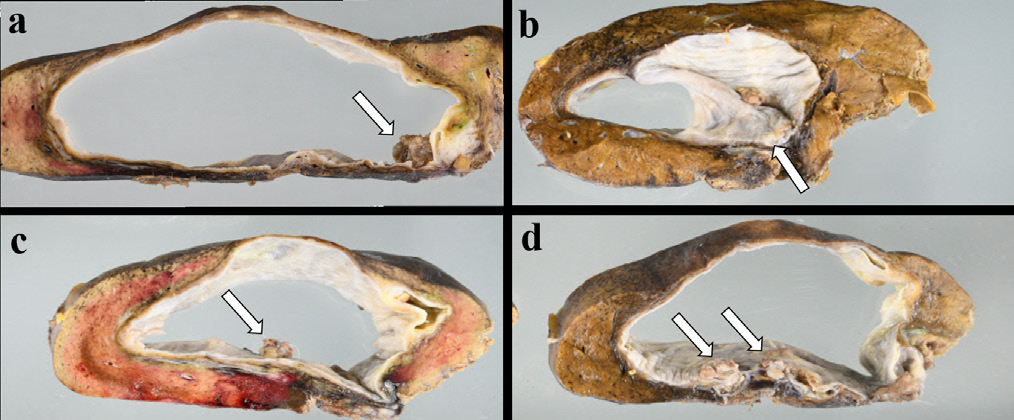
Macroscopic findings of resected specimens. (**a–d**) Mural nodules (white arrow) are observed as irregular mass inside the cyst

**Figure 5.**
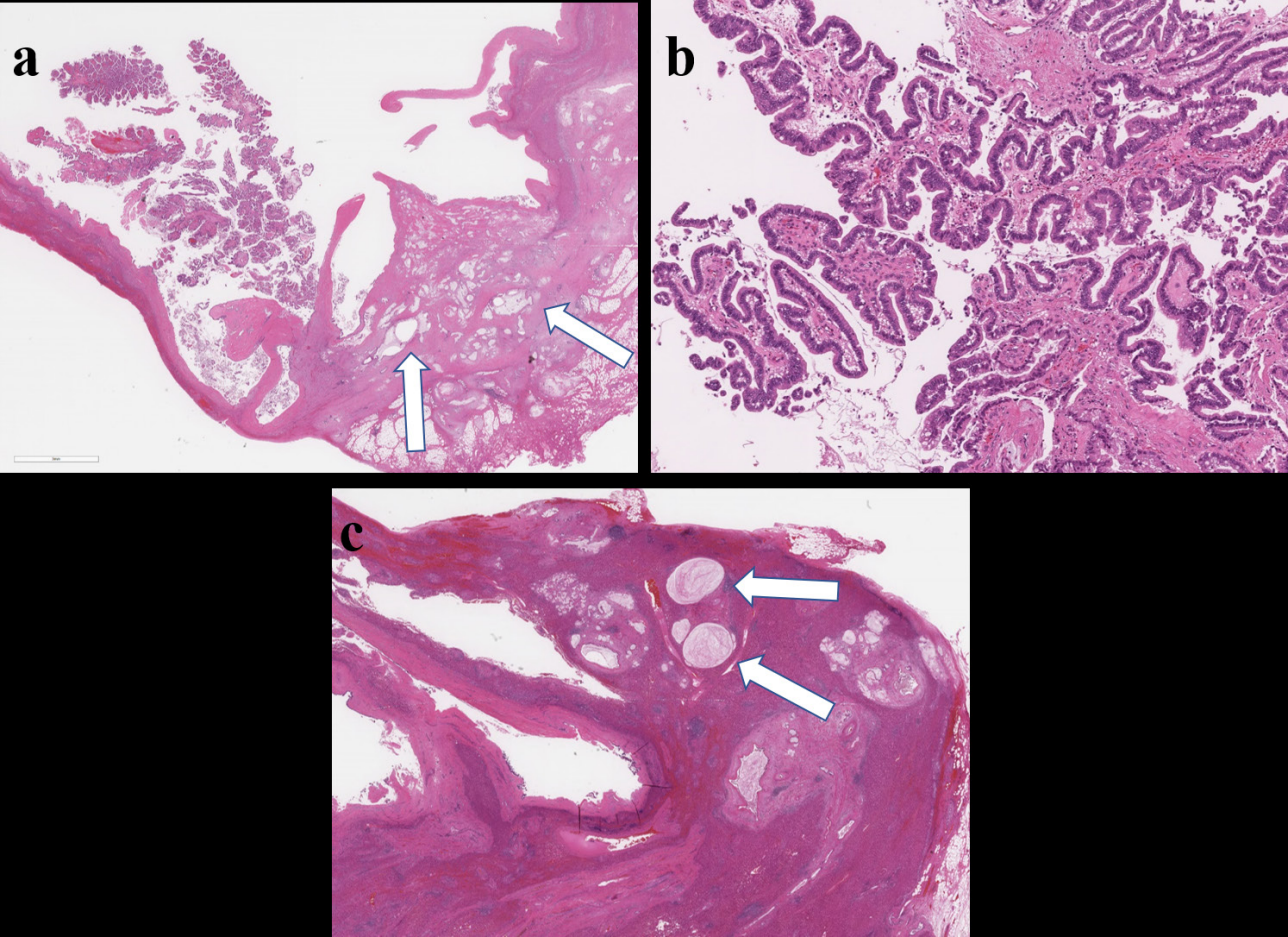
Microscopic findings of resected specimens. (**a**) Photomicrograph (low magnification; haematoxylin and eosin stain) shows the mural nodule invading the lesion of the liver (arrows). (**b**) A higher magnification view shows the mural nodule comprising of a papillary architecture in the mass. (**c**) Photomicrograph of the cyst wall (low magnification; haematoxylin and eosin stain) shows the mucin within the bile duct (arrows)

The pathological findings of the surgical specimen showed that the mural nodule with papillary proliferation of the atypical epithelium in the mass invaded the liver lesion (Figure 4, Figure 5). Several intrahepatic bile ducts were dilated with mucin, supporting the evidence of the mass being connected with the bile ducts. There was no ovarian-like interstitium in the mass. As a result of a thorough investigation, our patient was diagnosed with IPNB with an associated invasive carcinoma. Additional pathological examination of lymph nodes of the resection specimen showed metastasis, and pathological TNM stage (Unio Internationalis Contra Cancrum, eighth) was diagnosed as pT3N1MX.

## Discussion

The number of diagnosed hepatic cystic masses is increasing as a result of the rapid advancements in imaging studies. Currently, it has been estimated that 20% of the general population has cystic masses in the liver.^1^ While the majority of these masses are benign simple cysts, 3–5% of diagnosed cases may represent biliary cystic tumours.^1^ Furthermore, 20–30% of patients with diagnosed cases of hepatic cystic masses are at risk of their malignant transformation.^1^

The most common bile duct cystic tumours are IPNB and MCN neoplasms. IPNB was found in 10% of all resected bile duct tumours.^2^ Out of all diagnosed IPNB cases, approximately 40–80% presented with invasive carcinoma, or tubular, or mucinous adenocarcinoma.^3^ These findings suggest the high malignancy potential of IPNB. In contrast, MCN is a rare disease with a reported incidence of less than 5% of all hepatic cystic tumours.^4^ Additionally, only 20% of all diagnosed MCN cases are at risk of developing malignancy.^5^

IPNB tends to grow by connecting to the bile ducts compared with MCN. This is considered the imaging differentiation point between MCN and IPNB.^6^

IPNB and MCN are described in the independent chapters in the fifth edition of the World Health Organization (WHO) series on the classification of human tumours, published in 2019,^7^ but there are only a few studies mentioned because both IPNB and MCN are relatively new disease concepts.

In general, IPNB is classified as IPNB without mucin secretion (IPNB-NM) and IPNB with mucin secretion (IPMN-B). IPNB was classified by Ying et al into seven types based on morphology (Ⅰ, upstream-ductectatic type; II, typical type; Ⅲ, superficial-spreading type; IV, no-mass-forming type; V, intrahepatic-cystic type; Ⅵ, extrahepatic-cystic type; and Ⅶ, infiltrating type).^2^ Among the 68 IPMN-B patients, type II was the most common (30 patients), followed by type V (13 patients) and type IV (11 patients). Among the 13 IPNB-NM patients, type I was the most common (11 patients), followed by type II (one patient) and type VII (one patient).

Ying et al also showed that IPNB-NM mainly manifests as type I because it does not cause mucus secretion.^2^ In contrast, IPMN-B mostly presents as types Ⅱ–Ⅶ because it promotes mucin accumulation, where mucin is secreted in the bile ducts, subsequently causing aggravation of the bile ducts dilation. The case we experienced was classified as IPMN-B type V, which is not typical but is relatively frequent. Additionally, it is consistent with the features of IPMN-B showed by Ying et al.

The imaging findings of IPNB are mucobilia, dilated ducts, enhancement and the metabolic activity of mural nodules. The majority of malignant IPNB presentations have wall nodules with contrast enhancement or solid components that infiltrate the adjacent liver parenchyma. They may appear as a small lesion or focal thickening of the wall leading up to the lesion.^8^ In these cases, the findings are consistent with the malignant IPNB.

Previously, IPNB was considered as a counterpart of the pancreatic intraductal papillary mucinous neoplasm.^9^ In the fifth edition of the WHO’s classification of human tumours, revised in 2019, IPNB was defined as a grossly visible premalignant neoplasm with intraductal papillary or villous growth of biliary-type epithelium. In addition, IPNB has been classified as low, intermediate and high grade in the fourth edition of the WHO’s classification of human tumours, revised in 2010.^10^ However, in the fifth edition, IPNB has been classified as low grade, high grade and an associated invasive carcinoma.^7^ As this indicates that IPNB is mostly recognized as a precancerous lesion, this case report emphasizes the importance of capturing the image findings of IPNB to aid in early diagnosis and timely resection and improvement of IPNB prognosis. It is expected that more cases of IPNB will be reported in the future as a well-established imaging disease, just as it is in pathology.

## Learning points

1. IPNB is a relatively rare disease that should be considered in the differential diagnosis of hepatic cystic tumours.
2. Radiologists should be aware of the importance of capturing image findings of IPNB as this disease has a high potential for malignancy.
